# Molecular Imaging of Vascular Calcification with ^18^F-Sodium-Fluoride in Patients Infected with Human Immunodeficiency Virus

**DOI:** 10.3390/ijms20051183

**Published:** 2019-03-08

**Authors:** Paolo Raggi, Napoleone Prandini, Guido Ligabue, Giovanni Braglia, Francesco Esposito, Jovana Milic, Andrea Malagoli, Riccardo Scaglioni, Giulia Besutti, Barbara Beghetto, Giulia Nardini, Enrica Roncaglia, Cristina Mussini, Giovanni Guaraldi

**Affiliations:** 1 Division of Cardiology and Mazankowski Alberta Heart Institute, University of Alberta, Edmonton, 11220 83rd Avenue, Suite 5A9-014, Edmonton, AB T6G 2B7, Canada; 2 Department of Nuclear Medicine, Azienda Ospedaliero-Universitaria di Modena; University of Modena and Reggio Emilia, 41124 Modena, Italy; prandini.napoleone@policlinico.mo.it; 3 Department of Radiology, Azienda Ospedaliero-Universitaria di Modena; University of Modena and Reggio Emilia, 41124 Modena, Italy; ligabue.guido@unimore.it (G.L.); riccardo.scaglioni@hotmail.it (R.S.); 4 Modena HIV Metabolic Clinic, Azienda Ospedaliero-Universitaria di Modena; University of Modena and Reggio Emilia, 41124 Modena, Italy; giovannibraglia92@libero.it (G.B.); francescoesposito93@gmail.com (F.E.); jovana.milic@gmail.com (J.M.); andrea.malagoli@gmail.com (A.M.); cristina.mussini@unimore.it (C.M.); giovanni.guaraldi@unimore.it (G.G.); 5 Clinical and Experimental Medicine PhD Program, University of Modena and Reggio Emilia, 41124 Modena, Italy; giulia.besutti@libero.it (G.B.); beghetto.barbara@policlinico.mo.it (B.B.); giulia.nardini@unimore.it (G.N.); roncaglia.enrica@policlinico.mo.it (E.R.)

**Keywords:** immune deficiency syndrome, sodium fluoride, atherosclerosis, calcification

## Abstract

^18^F-Sodium Fluoride (NaF) accumulates in areas of active hydroxyapatite deposition and potentially unstable atherosclerotic plaques. We assessed the presence of atherosclerotic plaques in 50 adult patients with HIV (HIV+) who had undergone two cardiac computed tomography scans to measure coronary artery calcium (CAC) progression. CAC and its progression are predictive of an unfavorable prognosis. Tracer uptake was quantified in six arterial territories: aortic arch, innominate carotid artery, right and left internal carotid arteries, left coronary (anterior descending and circumflex) and right coronary artery. Thirty-one patients showed CAC progression and 19 did not. At least one territory with high NaF uptake was observed in 150 (50%) of 300 arterial territories. High NaF uptake was detected more often in non-calcified than calcified areas (68% vs. 32%), and in patients without than in those with prior CAC progression (68% vs. 32%). There was no correlation between clinical and demographic variables and NaF uptake. In clinically stable HIV+ patients, half of the arterial territories showed a high NaF uptake, often in the absence of macroscopic calcification. NaF uptake at one time point did not correlate with prior progression of CAC. Prospective studies will demonstrate the prognostic significance of high NaF uptake in HIV+ patients.

## 1. Introduction

The survival of HIV infected (HIV+) patients has increased significantly over the past several decades although it is still far from that of the general population [1]. However, the improved survival has been paralleled by a rise in the incidence of premature cardiovascular events, and cardiovascular disease is now among the leading causes of morbidity and mortality in HIV+ patients [2,3]. Atherosclerosis is the result of multiple contributing causes that include a higher prevalence of traditional risk factors, the HIV virus and other viral associated infections, as well as antiretroviral medications and their attendant metabolic alterations [4]. Molecular imaging offers an opportunity to image atherosclerosis in its early stages using probes that visualize activity within the plaque, taking advantage of specific mechanisms inherent in plaque formation. Positron emission tomography (PET) imaging with ^18^F-sodium fluoride (NaF) has been used for several decades to detect bone malignancies since this radiotracer adheres with high affinity on the surface of growing crystals of hydroxyapatite [5]. Active calcification is an integral part of atherosclerosis and initial publications reported visualization of atherosclerotic lesions in the carotid arteries and aorta of patients undergoing PET-computed tomography (PET-CT) imaging for oncological diagnostic purposes [6]. Subsequent publications highlighted the association of NaF with risk factors for atherosclerosis, risk scoring algorithms such as the Framingham risk score and even plaques with morphological features of instability on CT angiography and intraluminal coronary imaging [7,8,9,10]. In a seminal publication, Joshi et al. [11] showed that NaF accumulated preferentially in the culprit plaques of patients with a recent cerebrovascular event or acute coronary syndromes. Two groups of investigators reported that high NaF activity in the aortic and mitral valve leaflets at baseline predicted the development of valvular calcification and dysfunction during follow-up [12,13]. Hence, NaF may be an excellent marker of atherosclerotic disease activity and an indicator of the propensity to develop cardiovascular calcification, a predictor of high-risk of events in the general population. Therefore, we hypothesized that NaF imaging will detect ongoing and more prominent arterial osteogenesis in patients with significant progression of coronary artery calcium (CAC) in prior sequential CT scans of the chest. Hence, our objective was to assess the relationship between prior CAC progression and current NaF activity in several arterial beds.

## 2. Results

**Figure 1:**
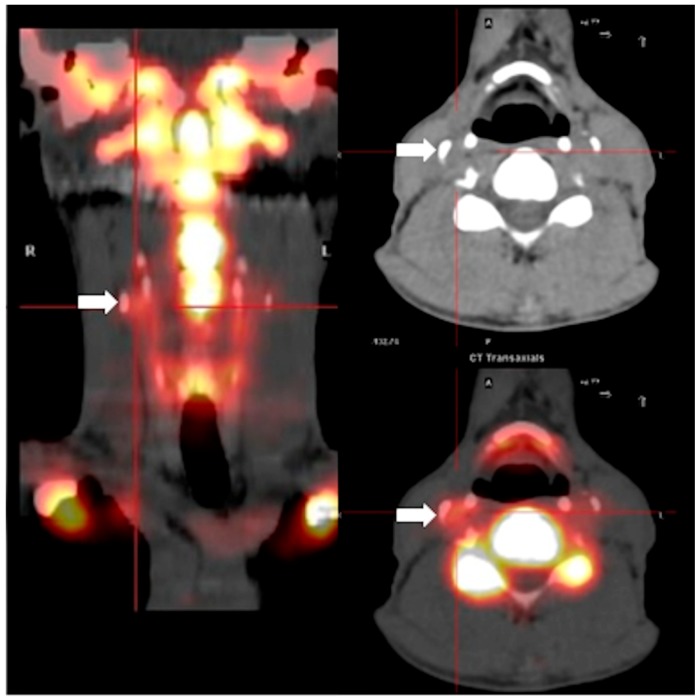
Example of carotid artery ^18^F-Sodium Fluoride uptake in a 50 years old patient with HIV. The image on the left is a coronal reconstrution of the PET image of the neck and base of the skull of this patient. The white arrow points at one of several areas of enhanced ^18^F-Sodium Fluoride uptake along the course of the right internal carotid artery. The image on the top right is an axial computed tomography section of the neck of the same patient, and the white arrow points at a calcified lesion corresponding to the lesion seen on the PET image on the left. The bottom right image is a fusion of axial PET and CT images; the white arrow points again at the same lesion shown in the axial CT image above, demonstrating ^18^F-Sodium Fluoride uptake in correspondence with the calcification.

**Figure 2:**
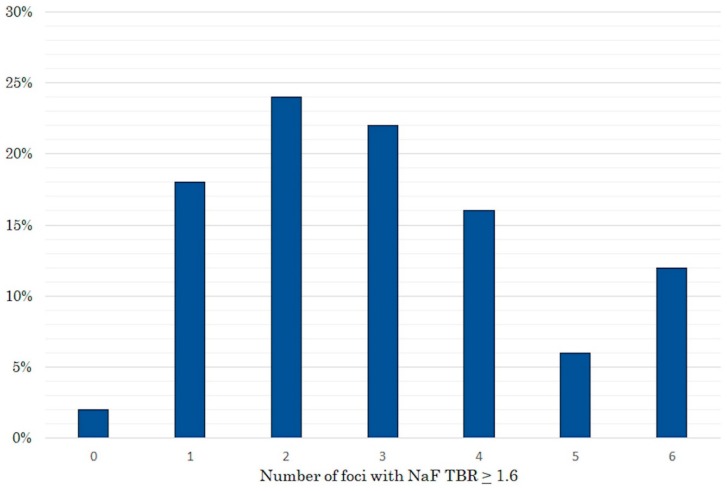
Proportion of patients showing high ^18^F-Sodium Fluoride uptake in 6 arterial territories. Only one patient had no uptake while 80% of the patients showed enhanced uptake in 1–4 territories.

Fifty adult HIV+ patients (84% men, mean age 57 ± 8 years) were enrolled. According to the predefined study methodology, 31 patients were classified as having had CAC progression and 19 as not having had CAC progression prior to NaF imaging. Table 1 summarizes the patients’ clinical characteristics based on CAC progression or non-progression. Few characteristics differentiated patients with CAC progression from those without: there were more men among patients with progression, patients were older, had a higher baseline CAC score, had a more frequent history of hypertension and the total and LDL cholesterol as well as the CD4 nadir count were significantly lower. Half of the patients had at least one area of high NaF uptake (Figure 1) in each of the six arterial territories: 20 had a high uptake in the right and 24 in the left carotid artery; 20 had a high uptake in the innominate artery; 25 had a high NaF uptake in the aortic arch. Finally, 24 patients had a high uptake in the right and 37 in the left coronary artery. In total, 150 of 300 (50%) arterial territories showed at least one area of high NaF uptake. Figure 2 shows the distribution -expressed as percentage of patients- of high NaF uptake in 0 to 6 arterial territories. Only one patient had no high NaF uptake in any territory, while most patients showed involvement of 1–4 territories. 

**Figure 3:**
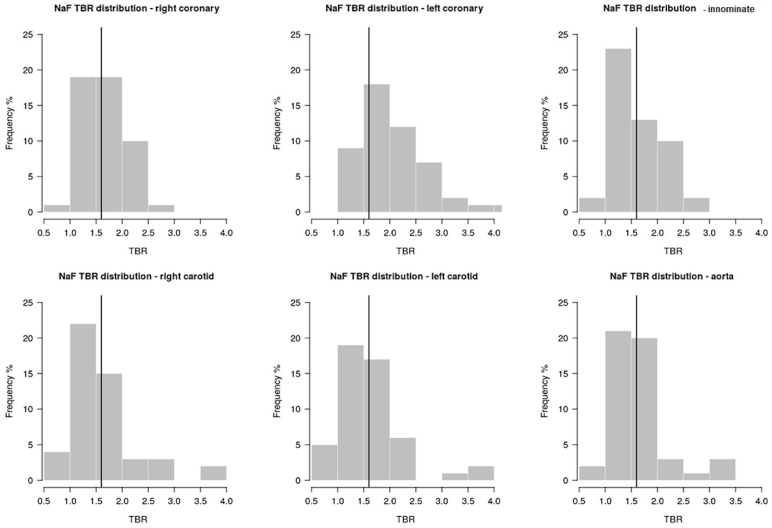
Proportional distribution of ^18^F-Sodium Fluoride uptake assessed as target-to-background ratio in each arterial territory. The vertical line identifies a target-to-background ratio of 1.6 that was chosen as a threshold for high ^18^F-Sodium Fluoride uptake.

The average TBR in patients with and without prior CAC progression was similar in all arterial territories (Table 1). Figure 3 shows the distribution of NaF uptake assessed as TBR in each arterial territory. NaF uptake was detected 93 times (62%) in areas without CAC and 57 times (38%) in areas with CAC. A high NaF arterial uptake was observed more frequently in patients who had had no CAC progression between scans (68%), than in patients with prior CAC progression (32%). The Spearman correlation coefficient between no prior progression of CAC and high NaF uptake was -0.56 (*p* = 0.01). Conversely, the Spearman correlation coefficient between prior CAC progression and NaF uptake was 0.20 (*p* = 0.33). There was no correlation between any clinical, laboratory and demographic variable and NaF uptake. Multivariable analyses did not identify any variable associated with a high NaF uptake in any of the arterial sites.

## 3. Discussion

Our findings are novel, as NaF imaging has never been attempted before in HIV+ patients. Calcification is a process inherent in atherosclerotic plaque formation and it proceeds through active mechanisms resembling bone formation; as NaF accumulates in areas of microcalcification, it is considered an optimal tracer to detect plaque growth. We described a NaF uptake prevalence of 50% in six arterial territories of ambulatory, stable patients infected with HIV. There was no correlation between prior CAC progression and active vascular osteogenesis at a later time point. Several areas of NaF uptake at the time of PET imaging did not correspond with macroscopic areas of calcification on CT, and NaF uptake was not associated with any clinical or demographic characteristic of the patients enrolled. 

Our main intention in designing this study was to investigate whether a significant prior progression of CAC is associated with active, ongoing osteogenesis in the vessel wall. Progression of CAC has been reported numerous times to be associated with adverse outcomes [14,15,16]; hence, it appears legitimate to wonder if the unfavourable outcome is related to ongoing wall damage. The prevalence of NaF uptake was high for the type of patients we studied; these were patients in stable clinical conditions, on a stable medical regimen and under good metabolic control. It may appear contradictory that NaF uptake was seen more frequently in patients without than with prior CAC increase, and that no clinical or laboratory variable was associated with NaF uptake. However, a few considerations may clarify some of these apparent paradoxes. First, a lack of power or unknown confounders may have determined the outcome of the statistical analyses, although other explanations may also apply. Only a minority of patients was receiving statins and this may have favored the development of new atherosclerosis foci actively accruing microcalcifications. Although none of the markers of HIV infection and risk factors for atherosclerosis were associated with NaF uptake, this is not entirely surprising. In fact, it has been reported numerous times in the general population, where CAC imaging has been implemented to identify asymptomatic atherosclerosis and assess risk of events [17,18], that as many as 50% of the patients with risk factors have no CAC, and as many as 30% of the subjects without risk factors have CAC [19]. In HIV+ positive patients, a similar concept is reflected in the lack of a strong association between risk factors, risk scoring algorithms and presence of subclinical atherosclerosis or occurrence of events [20]. Even adding HIV-specific factors to risk algorithms does not increase the ability to predict risk significantly [21]. What other factors can then induce development of atherosclerosis in these patients? And how can we better predict the occurrence of events? HIV is a state of chronic low-grade inflammation, and immune activation as much as immune suppression [22]. The pathological processes occurring at the level of the vessel wall may be beyond prediction and detection based on laboratory parameters and clinical characteristics alone. Hence, imaging may provide a means to fill the gap between clinical evidence and actual risk. In the general population, Marnane et al. [23] showed that high ^18^F-fluoro-deoxy-glucose (FDG) uptake in the carotid arteries predicted stroke recurrence. Similarly, Figeroa et al. [24], in a retrospective analysis of oncological patients submitted to FDG imaging, showed that the ascending aorta TBR was highly predictive of CV events at the end of a median follow-up of 4.2 years. Lee et al. [10] showed that NaF localizes in plaques with vulnerable features on computed tomography angiography and predicts the occurrence of acute coronary events and revascularization after two years of follow up. In HIV+ patients, studies are slowly accumulating to demonstrate that imaging may help improve risk prediction. For instance, Tawakol et al. [25] proved that arterial inflammation assessed with FDG imaging is associated with high-risk plaques on CT angiography, while Raggi et al. [26] showed that CAC and epicardial adipose tissue, a source of inflammatory chemokines, are predictive of events in HIV+ patients. Imaging may also help detect unexpected trends in vascular health in HIV. For example, using FDG Zanni et al. [27] showed complete disappearance of systemic inflammation from lymphnodes and soft tissues in HIV+ patients treated with ART, but a simultaneous increase in vascular inflammation. This was attributed to the smoldering, low-grade inflammation and immune activation typical of patients receiving ART. In this light, the predominant uptake of NaF in arterial segments without macroscopic calcifications suggests that de-novo lesions are continuously being formed on the basis of ongoing smoldering inflammation. On the other hand, the lack of correlation between prior CAC progression and current NaF uptake may be due to the fact that older plaques have become quiescent. Of interest, in our study, the LDL cholesterol level was significantly lower among HIV+ patients with progression of CAC than in those without. This may be consistent with prior observations that aggressive LDL cholesterol lowering is associated with significant CAC progression over time, likely representing a healing phenomenon of the plaque [28]. Only two prior publications have reported a prospective follow-up of arterial lesions imaged with a combination of NaF and CAC imaging. Ishiwata et al. [29] imaged 34 oncological and orthopedic patients at baseline and 1 year interval, and measured NaF uptake and CT calcium scores in the aorta and iliac arteries. Of 182 calcified sites, 96 showed NaF uptake. There was a very tenuous correlation between baseline mean NaF uptake and change in calcification over time. However, there was a significant correlation between maximum NaF uptake at baseline and progression of calcification at follow-up. In the experience reported by Li et al., [30] NaF and FDG uptake and CT calcium imaging in eight arterial districts were performed at baseline and after one year in 11 patients affected by multiple myeloma. The highest NaF at baseline was localized on the least calcified or non-calcified arterial territories, and these were the ones that showed most often an avid accumulation of NaF and FDG during follow-up. In contrast, NaF accumulated less often and less intensely both at baseline and during follow-up in areas of intense calcification. These observations once again support the view that densely calcified arterial lesions may be quiescent and mostly healed areas of atherosclerosis. 

Our study had some limitations. The sample size was relatively small and was composed predominantly of men. The assessment of CAC progression was done prior to performing NaF imaging and this did not allow us to assess change in a prospective manner. These are very preliminary observations and no outcome data are available to validate the prognostic utility of NaF in the HIV+ population. There is currently no known threshold for NaF uptake that defines high vs low risk; therefore, we used a threshold similar to that reported in prior publications [11,31] but we have no way to know at the current state of knowledge if that threshold was too low or too high.

## 4. Materials and Methods

The study was approved by the Health Research Ethics Board at the University of Alberta (Pro00058107; July 17, 2015) and the Comitato Etico Provinciale of the University of Modena and Reggio Emilia (Protocol N. 1471, April 20, 2016). All patients signed an informed consent to participate in the study that was conducted according to the Declaration of Helsinki for research in human subjects (https://www.wma.net/policies-post/wma-declaration-of-helsinki-ethical-principles-for-medical-research-involving-human-subjects/).

### 4.1. Patients Selection

We enrolled 50 consecutive HIV+ patients from the Modena HIV Metabolic Clinic (MHMC), treated for at least 6 months with stable doses of anti-retroviral therapeutic (ART) agents prior to enrolment. MHMC is a multidisciplinary center for the diagnosis and treatment of non-infectious co-morbidities in HIV-infected patients. Patients are assessed for lipodystrophy, diabetes mellitus, systemic hypertension, risk and history of cardiovascular disease (CVD), osteoporosis, kidney failure, liver and lung diseases through a comprehensive clinical evaluation and a select number of diagnostic tests, including dual energy X-ray absorptiometry (DEXA), chest and abdominal CT scans and laboratory tests. The thoracic and abdominal CT scans are performed to assess and quantify coronary artery calcium (CAC), lung parenchyma abnormalities, visceral adipose tissue and liver steatosis. 

To be included in the present study, patients had to have previously undergone 2 CT scans a minimum of one year apart to assess CAC progression. All patients were ambulatory and were receiving optimal medical therapy with stable doses (at least six months without changes) of ART, oral or injectable hypoglycemic agents, lipid lowering and blood pressure lowering drugs. Information on medical history, risk factors for atherosclerotic heart disease, and medications was collected via questionnaire and further clarified by review of the patients’ medical records. 

Laboratory test results obtained within 90 days of PET/CT imaging were collected from the electronic health records of the patients enrolled. The estimated glomerular filtration rate (eGFR) was calculated using the CKD-Epi equation [32]. The 10-year risk of cardiovascular events was calculated using the Pooled Equations formula according to the 2013 American College of Cardiology and American Heart Association guidelines [33]. 

### 4.2. HIV Related History and Immuno-Virological Parameters

We recorded the plasma HIV-1 RNA levels, cumulative exposure to integrase strand transfer inhibitors (INSTI), non-nucleoside reverse transcriptase inhibitors (NNRTI), nucleoside reverse transcriptase inhibitors (NRTI) and protease inhibitors (PI). An undetectable serum HIV viral load was defined as < 40 copies/mL. Previous AIDS diagnosis was defined according to the Centers for Disease Control group “C” category. Immunological parameters of HIV infection were T-helper lymphocyte (CD4+) nadir, and current CD4+ and CD8+ count, as well the CD4+/CD8+ ratio. 

### 4.3. Imaging Acquisition and Interpretation

#### 4.3.1. Computer Tomography for Coronary Artery Calcium 

All patients underwent at least two consecutive gated CT scans of the chest at a minimal interval of 1 year and up to 2 years apart to measure changes in CAC. CT imaging was done with a Volume CT 64-slice CT scanner (GE Medical Systems, Milwaukee, WI), and was performed during a 20–30 sec breath hold with the following CT settings: 320 mAs and 140 Kv. Image acquisition was prospectively triggered at 80% of the R-R interval on the surface electrocardiogram and started at the level of the bronchial carina and extended to the diaphragm in sections 2.5 mm thick. To reconstruct the raw image data, we used a section thickness of 3.0 mm, a field of view of 20 cm^2^, and a matrix of 512 × 512, yielding a nominal pixel size of 0.39 mm^2^ and a voxel of 0.4 mm^3^. The CAC score was assessed using the “Smart Score” software (GE Medical Systems, Milwaukee, WI) on an off-line workstation. The CAC score was calculated according to the Agatston method, as previously described [34]. CAC progression was defined as a baseline CAC score = 0 followed by a CAC score >30, or a baseline CAC score >30 with a yearly progression >15%. These thresholds were derived from previous studies of CAC score reproducibility [35], and studies addressing the threshold of CAC score change associated with an adverse outcome [14,15].

#### 4.3.2. Positron Emission Tomography with ^18^F-Sodium-Fluoride

Patients received an injection of 370 MBq (10 mCi) of NaF and relaxed for 1 h before imaging. All examinations were performed on a PET/CT system (General Electric Discovery STE, Boston, MA, USA) with PET images obtained in 2 bed positions over the heart and aortic arch and cranial arteries (ECG-gated list-mode PET acquisition). A 16-slice multi-detector CT scanner was used to acquire matching data during quiet breathing (0.5 secs per rotation, 100 mAs tube current, 120 kVp tube voltage). CT data were reconstructed in 3 mm thick slices with no overlap. PET data were obtained in 3D mode and reconstructed with iterative reconstruction (RAMLA), in 3.75 mm isotropic voxels using CT-based attenuation correction. The global estimated radiation dose absorbed by each patient was between 4–6 mSv. The PET/CT and the last gated chest CT scan in the sequence of CAC scoring were performed within 1 month of each other. 

#### 4.3.3. Image Analysis 

All images were reviewed on an Xeleris workstation (GE Healthcare). The PET images were scaled with an upper standard uptake value (SUV) threshold of 2.0 and lower threshold of 0.0 for review. The PET images, CT images, and fused PET/CT images were reviewed synchronously in multiple planes. The following six arterial territories were independently evaluated: aortic arch, innominate carotid artery, right and left internal carotid arteries, left (anterior descending and circumflex combined) and right coronary artery. The SUV max was measured in the most NaF avid focus for each artery. For comparative blood pool measurements, we measured the SUV mean and SUV max of the superior vena cava and left ventricle (LV) blood pool. A 2-cm diameter region of interest was placed within the superior vena cava and the LV, avoiding myocardium, cardiac valves, and papillary muscles. The superior vena cava blood pool SUV was used to measure the target-to-background ratio (TBR) for the carotid arteries and aortic arch, while the LV SUV was used to calculate the coronary arteries TBR, as SUV max (coronary)/SUV mean (LV blood pool). As suggested in prior reports, a TBR of ≥ 1.6 was considered abnormal [29]. To limit the reproducibility error, we used only one reader for each of the imaging studies: NP interpreted all PET studies and GL all CAC scores; each one of these experienced readers used a semi-automatic software and the reproducibility was over 95%.

### 4.4. Statistical Analysis

The patients were divided in two groups depending on progression or non-progression of CAC scores between sequential CT scans. Descriptive analyses of demographic, clinical and imaging characteristics were performed based on CAC progression. As discussed, a TBR ≥ 1.6 was chosen to define high NaF uptake. Means (SD) of continuous, normally distributed variables were compared using the *t*-test. Medians of non-normally distributed continuous variables were compared using the Mann-Whitney test. Percentages of categorical variables were compared using the Χ^2^ test. Multivariable logistic regression was used to assess predictors of TBR > 1.6 using the following variables: age, sex, LDL-c, hemoglobin A1c, total Agatston score, eGFR, use of statins, duration of treatment with anti-retroviral medications, diabetes mellitus, and previous history of cardiovascular disease. The Fisher exact test and the Spearman correlation coefficient were used for statistical correlation of tracer uptake with progression and non-progression of CAC. Statistical significance was set at *p* < 0.05.

## 5. Conclusions

In summary, in 50 clinically stable and ambulatory HIV+ patients, we did not find a correlation between prior CAC progression and current NaF activity in several arterial beds. However, we detected a high (50%) prevalence of arterial NaF uptake. This is a remarkably higher prevalence than recently reported in another subset of patients at high-risk of cardiovascular disease in the general population. In fact, only 15% of ambulatory and stable patients with diabetes mellitus had evidence of high NaF uptake in the coronary arteries in that study [36]. This information suggests that patients living with HIV are prone to development of atherosclerosis, even when apparently clinically stable and metabolically well managed. The prognostic significance of these preliminary data will need to be confirmed in larger prospective studies. 

## Figures and Tables

**Table 1:** Patients’ clinical characteristics.

Variables	Total	No Progression	Progression	*p*-Value
	50	19(38%)	31(62%)	
**Demographic and anthropometric variables**				
Age, mean (±SD)	57.1 (7.82)	52.53 (4.64)	59.9 (8.1)	**<0.001**
Men (%)	42 (84%)	13 (68.42%)	29 (93.55%)	**0.05**
Pack year smoking, median (IQR)	15 (0–33.75)	16.88 (6.5–30.2)	13.2 (0–34.5)	0.88
BMI, kg/m^2^, mean (±SD)	26.01 (4.14)	25.66 (4.2)	26.21 (4.16)	0.67
Waist circumference, cm, mean (±SD)	96.78 (11.3)	96.81 (11.65)	96.76 (11.31)	0.99
**HIV variables**				
Nadir CD4 c/microL, median (IQR)	200 (107.5–324)	249.5 (142.75–430.5)	170 (59–286)	**0.02**
HIV duration, months, median (IQR)	277.5 (191.25–354)	267 (113–340)	287 (239.5–358.5)	0.19
CD4/CD8 ratio, mean (±SD)	0.97 (0.41)	1.01 (0.37)	0.94 (0.44)	0.60
Undetectable HIV viral load (%)	50 (100%)	19 (100%)	31 (100%)	1.0
Cumulative exposure to INSTIs, months, median (IQR)	36 (14–73.5)	28.5 (5.5–47.25) [12]	39 (23–98)	0.12
Cumulative exposure to NNRTIs, months, median (IQR)	63.5 (29.25–123.75)	39.5 (24–77.5) [10]	79.5 (29.75–126.5)	0.23
Cumulative exposure to NRTIs, months, median (IQR)	169 (103.5–235)	128 (55–196)	187.5 (136–251.75)	0.03
Cumulative exposure to PIs, months, median (IQR)	123 (51–172)	94.5 (50.75–136.5)	129 (51–188)	0.19
**Laboratory variables**				
CRP, mean (±SD)	0.24 (0.29)	0.22 (0.15)	0.25 (0.35)	0.57
Triglycerides, mg/dL, mean (±SD)	160.2 (160.51)	206.53 (231.96)	133.03 (92.68)	0.16
LDL cholesterol, mg/dL, mean (±SD)	103.26 (33.04)	124.71 (31.33)	90.69 (27.39)	**<0.001**
HDL cholesterol, mg/dL, mean (±SD)	46.13 (12.07)	45.59 (11.25)	46.45 (12.72)	0.82
Total cholesterol, mg/dL, mean (±SD)	173.15 (39.83)	198.88 (29.03)	158.07 (37.83)	**<0.001**
Glucose, mg/dL, mean (±SD)	103.26 (31.15)	95.06 (16.17)	108.34 (36.94)	0.30
HOMA, mean (±SD)	3.52 (4.24)	3.62 (3.06)	3.46 (4.97)	0.22
CKD-Epi, mL/min/1.73m^2^, mean (±SD)	79.77 (22.49)	85.73 (21.72)	76.33 (22.78)	0.28
**Comorbidities**				
CKD (%)	19 (38%)	5 (26.32%)	14 (45.16%)	0.30
COPD (%)	4 (8%)	0 (0%)	4 (12.9%)	0.27
Osteoporosis (%)	15 (30%)	4 (21.05%)	11 (35.48%)	0.44
Dyslipidemia (%)	49 (98%)	19 (100%)	30 (96.77%)	1
**Cardiovascular variables**				
Statin use (%)	7 (14%)	2 (10.53%)	5 (16.13%)	0.89
Systolic blood pressure, mmHg, mean (±SD)	127.25 (15.29)	121.94 (11.34)	130.43 (16.59)	0.08
Diastolic blood pressure, mmHg, mean (±SD)	81.58 (8.82)	81.33 (8.51)	81.73 (9.15)	0.88
ASCVD, mean (±SD)	10.31 (9.9)	7.39 (7.05)	13.59 (11.99)	0.21
Calcium score, median (IQR),	104 (38.5–348)	4 (2–6)	206 (104–490)	**0.05**
Hypertension (%)	33 (66%)	8 (42.11%)	25 (80.65%)	**0.01**
Type 2 diabetes mellitus (%)	14 (28%)	5 (26.32%)	9 (29.03%)	1
CVD (%)	7 (14%)	1 (5.26%)	6 (19.35%)	0.33
**Nuclear medicine variables**				
Aortic arch TBR	1.65 (0.52)	1.53 (0.51)	1.73 (0.52)	0.16
Innominate artery TBR	1.66 (0.49)	1.67 (0.57)	1.65 (0.45)	0.87
Right carotid artery TBR	1.69 (0.8)	1.59 (0.72)	1.75 (0.85)	0.42
Left carotid artery TBR	1.64 (0.66)	1.51 (0.69)	1.72 (0.63)	0.14
RCA TBR	1.65 (0.41)	1.6 (0.39)	1.67 (0.43)	0.55
LCA TBR	2.08 (0.66)	1.93 (0.43)	2.17 (0.76)	0.42

Legend: ASCVD: atherosclerotic cardiovascular disease risk score: BMI: body mass index. CKD: chronic kidney disease. COPD: chronic obstructive pulmonary disease. CRP: C-reactive protein. CVD: cardiovascular disease. HDL: high density lipoprotein. HOMA: homeostatic model assessment INSTI: integrase strand transfer inhibitors. LCA: left coronary artery. LDL: low density lipoprotein. NNRTI: non-nucleoside reverse transcriptase inhibitors. NRTI: nucleoside reverse transcriptase inhibitors. PI: protease inhibitors. RCA: right coronary artery. TBR: target to background ratio.

## References

[1] Hogg R.S., Eyawo O., Collins A.B., Zhang W., Jabbari S., Hull M.W., Lima V.D., Ahmed T., Kendall C.E., Althoff K.N. (2017). Health-adjusted life expectancy in HIV-positive and HIV-negative men and women in British Columbia, Canada: A population-based observational cohort study. Lancet HIV.

[2] Freiberg M.S., Chang C.-C.H., Kuller L.H., Skanderson M., Lowy E., Kraemer K.L., Butt A.A., Bidwell Goetz M., Leaf D., Oursler K.A. (2013). HIV infection and the risk of acute myocardial infarction. JAMA Intern. Med..

[3] Triant V.A., Grinspoon S.K. (2017). Epidemiology of ischemic heart disease in HIV. Curr. Opin. HIV AIDS.

[4] Shahbaz S., Manicardi M., Guaraldi G., Raggi P. (2015). Cardiovascular disease in human immunodeficiency virus infected patients: A true or perceived risk?. World J. Cardiol..

[5] Blau M., Nagler W., Bender M.A. (1962). Fluorine-18: A new isotope for bone scanning. J. Nucl. Med..

[6] Derlin T., Richter U., Bannas P., Begemann P., Buchert R., Mester J., Klutmann S. (2010). Feasibility of ^18^F-sodium fluoride PET/CT for imaging of atherosclerotic plaque. J. Nucl. Med..

[7] Derlin T., Wisotzki C., Richter U., Apostolova I., Bannas P., Weber C., Mester J., Klutmann S. (2011). In vivo imaging of mineral deposition in carotid plaque using ^18^F-sodium fluoride PET/CT: Correlation with atherogenic risk factors. J. Nucl. Med..

[8] Oliveira-Santos M., Castelo-Branco M., Silva R., Gomes A., Chichorro N., Abrunhosa A., Donato P., de Lima J.P., Pego M., Gonçalves L. (2017). Atherosclerotic plaque metabolism in high cardiovascular risk subjects—A subclinical atherosclerosis imaging study with ^18^F-NaF PET-CT. Atherosclerosis.

[9] Kitagawa T., Yamamoto H., Toshimitsu S., Sasaki K., Senoo A., Kubo Y., Tatsugami F., Awai K., Hirokawa Y., Kihara Y. (2017). (18)F-sodium fluoride positron emission tomography for molecular imaging of coronary atherosclerosis based on computed tomography analysis. Atherosclerosis.

[10] Lee J.M., Bang J.I., Koo B.K., Hwang D., Park J., Zhang J., Yaliang T., Suh M., Paeng J.C., Shiono Y. (2017). Clinical relevance of (18)F-sodium fluoride positron-emission tomography in noninvasive identification of high-risk plaque in patients with coronary artery disease. Circ. Cardiovasc. Imaging.

[11] Joshi N.V., Vesey A.T., Williams M.C., Shah A.S., Calvert P.A., Craighead F.H., Yeoh S.E., Wallace W., Salter D., Fletcher A.M. (2014). ^18^F-fluoride positron emission tomography for identification of ruptured and high-risk coronary atherosclerotic plaques: A prospective clinical trial. Lancet.

[12] Jenkins W.S., Vesey A.T., Shah A.S., Pawade T.A., Chin C.W., White A.C., Fletcher A., Cartlidge T.R., Mitchell A.J., Pringle M.A. (2015). Valvular (18)F-Fluoride and (18)F-Fluorodeoxyglucose Uptake Predict Disease Progression and Clinical Outcome in Patients With Aortic Stenosis. J. Am. Coll. Cardiol..

[13] Massera D., Trivieri M.G., Andrews J.P.M., Sartori S., Abgral R., Chapman A.R., Jenkins W.S.A., Vesey A.T., Doris M.K., Pawade T.A. (2019). Disease Activity in Mitral Annular Calcification. Circ. Cardiovasc. Imaging.

[14] Raggi P., Callister T.Q., Shaw L.J. (2004). Progression of coronary artery calcium and risk of first myocardial infarction in patients receiving cholesterol-lowering therapy. Arterioscler. Thromb. Vasc. Biol..

[15] Budoff M.J., Hokanson J.E., Nasir K., Shaw L.J., Kinney G.L., Chow D., Demoss D., Nuguri V., Nabavi V., Ratakonda R. (2010). Progression of coronary artery calcium predicts all-cause mortality. JACC Cardiovasc. Imaging.

[16] Budoff M.J., Young R., Lopez V.A., Kronmal R.A., Nasir K., Blumenthal R.S., Detrano R.C., Bild D.E., Guerci A.D., Liu K. (2013). Progression of coronary calcium and incident coronary heart disease events: MESA (Multi-Ethnic Study of Atherosclerosis). J. Am. Coll. Cardiol..

[17] Mortensen M.B., Falk E., Li D., Nasir K., Blaha M.J., Sandfort V., Rodriguez C.J., Ouyang P., Budoff M. (2018). Statin trials, cardiovascular events, and coronary artery calcification: Implications for a trial-based approach to statin therapy in MESA. JACC Cardiovasc. Imaging.

[18] Gibson A.O., Blaha M.J., Arnan M.K., Sacco R.L., Szklo M., Herrington D.M., Yeboah J. (2014). Coronary artery calcium and incident cerebrovascular events in an asymptomatic cohort. The MESA Study. JACC Cardiovasc. Imaging.

[19] Nasir K., Bittencourt M.S., Blaha M.J., Blankstein R., Agatson A.S., Rivera J.J., Miedema M.D., Sibley C.T., Shaw L.J., Blumenthal R.S. (2015). Implications of coronary artery calcium testing among statin candidates according to American College of Cardiology/American Heart Association cholesterol management guidelines: MESA (Multi-Ethnic Study of Atherosclerosis). J. Am. Coll. Cardiol..

[20] Feinstein M.J., Nance R.M., Drozd D.R., Ning H., Delaney J.A., Heckbert S.R., Budoff M.J., Mathews W.C., Kitahata M.M., Saag M.S. (2017). Assessing and refining myocardial infarction risk estimation among patients with human immunodeficiency virus: A study by the centers for AIDS Research Network of Integrated Clinical Systems. JAMA Cardiol..

[21] Raggi P., De Francesco D., Manicardi M., Zona S., Bellasi A., Stentarelli C., Carli F., Beghetto B., Mussini C., Malagoli A. (2016). Prediction of hard cardiovascular events in HIV patients. J. Antimicrob. Chemother..

[22] Triant V.A. (2013). Cardiovascular disease and HIV infection. Curr. HIV/AIDS Rep..

[23] Marnane M., Merwick A., Sheehan O.C., Hannon N., Foran P., Grant T., Dolan E., Moroney J., Murphy S., O’Rourke K. (2012). Carotid plaque inflammation on ^18^F-fluorodeoxyglucose positron emission tomography predicts early stroke recurrence. Ann. Neurol..

[24] Figueroa A.L., Abdelbaky A., Truong Q.A., Corsini E., MacNabb M.H., Lavender Z.R., Lawler M.A., Grinspoon S.K., Brady T.J., Nasir K. (2013). Measurement of arterial activity on routine FDG PET/CT images improves prediction of risk of future CV events. JACC Cardiovasc. Imaging.

[25] Tawakol A., Lo J., Zanni M.V., Marmarelis E., Ihenachor E.J., MacNabb M., Wai B., Hoffmann U., Abbara S., Grinspoon S. (2014). Increased arterial inflammation relates to high-risk coronary plaque morphology in HIV-infected patients. J. Acquir. Immune Defic. Syndr..

[26] Raggi P., Zona S., Scaglioni R., Stentarelli C., Ligabue G., Besutti G., Menozzi M., Santoro A., Malagoli A., Bellasi A. (2015). Epicardial adipose tissue and coronary artery calcium predict incident myocardial infarction and death in HIV-infected patients. J. Cardiovasc. Comput. Tomogr..

[27] Zanni M.V., Toribio M., Robbins G.K., Burdo T.H., Lu M.T., Ishai A.E., Feldpausch M.N., Martin A., Melbourne K., Triant V.A. (2016). Effects of antiretroviral therapy on immune function and arterial inflammation in treatment-naive patients with human immunodeficiency virus infection. JAMA Cardiol..

[28] Henein M., Granåsen G., Wiklund U., Schmermund A., Guerci A., Erbel R., Raggi P. (2015). High dose and long-term statin therapy accelerate coronary artery calcification. Int. J. Cardiol..

[29] Ishiwata Y., Kaneta T., Nawata S., Hino-Shishikura A., Yoshida K., Inoue T. (2017). Quantification of temporal changes in calcium score in active atherosclerotic plaque in major vessels by (18)F-sodium fluoride PET/CT. Eur. J. Nucl. Med. Mol. Imaging.

[30] Li X., Heber D., Cal-Gonzalez J., Karanikas G., Mayerhoefer M.E., Rasul S., Beitzke D., Zhang X., Agis H., Mitterhauser M. (2017). Association between osteogenesis and inflammation during the progression of calcified plaque evaluated by (18)F-Fluoride and (18)F-FDG. J. Nucl. Med..

[31] Irkle A., Vesey A.T., Lewis D.Y., Skepper J.N., Bird J.L., Dweck M.R., Joshi F.R., Gallagher F.A., Warburton E.A., Bennett M.R. (2015). Identifying active vascular microcalcification by (18)F-sodium fluoride positron emission tomography. Nat. Commun..

[32] Levey A.S., Stevens L.A., Schmid C.H., Zhang Y.L., Castro A.F., Feldman H.I., Kusek J.W., Eggers P., Van Lente F., Greene T. (2009). A new equation to estimate glomerular filtration rate. Ann. Intern. Med..

[33] Goff D.C., Lloyd-Jones D.M., Bennett G., Coady S., D’Agostino R.B., Gibbons R., Greenland P., Lackland D.T., Levy D., O’Donnell C.J. (2014). 2013 ACC/AHA guideline on the assessment of cardiovascular risk: A report of the American College of Cardiology/American Heart Association Task Force on Practice Guidelines. Circulation.

[34] Agatston A.S., Janowitz W.R., Hildner F.J., Zusmer N.R., Viamonte M., Detrano R. (1990). Quantification of coronary artery calcium using ultrafast computed tomography. J. Am. Coll. Cardiol..

[35] Callister T.Q., Cooil B., Raya S.P., Lippolis N.J., Russo D.J., Raggi P. (1998). Coronary artery disease: Improved reproducibility of calcium scoring with an electron-beam CT volumetric method. Radiology.

[36] Raggi P., Senior P., Shahbaz S., Kaul P., Hung R., Coulden R., Yeung R., Abele J. (2019). (18)F-sodium fluoride imaging of coronary atherosclerosis in ambulatory patients with diabetes mellitus. Arterioscler. Thromb. Vasc. Biol..

